# Whole-genome sequence of *Bacillus velezensis* strain R22 isolated from *Oryza sativa* rhizosphere in Bulgaria

**DOI:** 10.1128/MRA.00693-23

**Published:** 2023-11-28

**Authors:** Penka Petrova, Maria Gerginova, Alexander Arsov, Nadya Armenova, Lidia Tsigoriyna, Emanoel Gergov, Kaloyan Petrov

**Affiliations:** 1 Institute of Microbiology, Bulgarian Academy of Sciences, Sofia, Bulgaria; 2 Institute of Chemical Engineering, Bulgarian Academy of Sciences, Sofia, Bulgaria; University of Strathclyde, Glasgow, United Kingdom

**Keywords:** *Bacillus velezensis*, antifungal activity, velezensin

## Abstract

*Bacillus velezensis* R22 was isolated from a rice rhizosphere in Bulgaria. Its genome (assembled into 14 scaffolds) has a size of 4.08 Mbp and a G + C content of 46.35%. Nine full biosynthetic clusters for antimicrobials were predicted, among them two new gene clusters probably encoding polyketides named macrolactin R22 and velezensin.

## ANNOUNCEMENT

*Bacillus velezensis* strains are considered the best green alternative to chemical fungicides and crop fertilizers (1–4). Many of them isolated from plant rhizosphere have been reported to show high antifungal activity against fungal plant pathogens due to lipopeptide synthesis (5–7).

*B. velezensis* R22 was isolated from a rice rhizosphere clay soil sample (less than 0.5 cm to the root) taken from the field of the Upper Thracian Lowland in Bulgaria (42.08 N 24.44 E). A suspension of 1 g of soil in 100 mL saline was shaken at 100 rpm and room temperature for 2 h, filtered through Whatman filter paper Grade 4, then plated on Thermo Scientific Oxoid Nutrient Broth No. 2 (CM0067B) supplemented with 1.5% agar, and incubated in aerobic conditions at 37°C for 24 h. The axenic culture was obtained from a single colony streaked on an agar plate three consecutive times. The strain was grown in Nutrient Broth No. 2 (Oxoid, UK) at 37°C, 160 rpm agitation for 24 h.

Genomic DNA for whole genome sequencing (WGS) was extracted with the GeneMATRIX Kit (EURx, Poland). The TruSeq DNA PCR-Free Kit (Illumina Inc., USA) was used for library construction. The sequencing was performed by Macrogen Inc. (Korea), using Illumina HiSeq 2000 System, read length 151. Total reads were 2,065,832; 75× genome coverage. Low-quality reads were trimmed using Trimmomatic version 0.36 (8). FastQC v0.11.5 (9) showed >96.8% reads with Q20. SPAdes v3.13.0 software (10) was used for the genome assembly, while the scaffolding was completed by Platanus v2.2.2 (11). All software was used with default parameters unless otherwise specified. The assembled genome was validated by BUSCO v5.4.7 (12) using lineage “bacteria_odb10.” Complete and single-copy BUSCOs were 124 (100%), while fragmented and missing BUSCOs were 0.0%.

The genome of *B. velezensis* strain R22 was assembled into 14 scaffolds with N_50_ value of 2,132,726 bp. It is 4,081,504  bp in size and has GC content of 46.35%. CheckM v1.2.2 showed 98.82% genome completeness and 0% contamination (13). NCBI Prokaryotic Genome Annotation Pipeline version 6.5 (14) predicted 4,087 genes encoding 3,935 proteins, 72 tRNAs, 14 rRNAs, and 5ncRNAs. The closest type strain genome is GCA_001461825.1 of *B. velezensis* NRRL B-41580T possessing 98.26% identity estimated by ANI, https://www.ezbiocloud.net/tools/ani (15), and 84.4% dDDH, calculated by TYGS, https://tygs.dsmz.de (16).

The analysis with antiSMASH v7.0, set at relaxed detection of secondary metabolites (17), showed that about 10% (340 kb) of the genome of *B. velezensis* R22 is devoted to the potential synthesis of non-ribosomal antimicrobial secondary metabolites: fengycin, bacillibactin, surfactin, macrolactin H, bacillaene, difficidin, and bacilysin. Two new gene clusters potentially encoding antibiotics were proposed. The first, found in Contig 4, contains type III polyketide synthase (PK) (18) and possibly encodes a novel polyketide named macrolactin R22. It shows 40%–44% similarity to the clusters encoding the polyketide macrolactin H in other rhizobacteria (19) but also 17% similarity with the myxovirescin cluster in *Myxococcus xanthus* (CP000113.1) (20). Another cluster, part of Contig 5 (30,123 bp), may encode a novel polyketide named velezensin with core biosynthetic genes type I PK and *trans* acyltransferase PK.

## Data Availability

This Whole Genome Shotgun project has been deposited in DDBJ/ENA/GenBank under accession no. JASUZW000000000.1, BioSample SAMN35683875. The version described in this paper is the first version. The Sequence Read Archive is deposited under accession no SRP441980.
